# Association Between Gastroesophageal Reflux Symptoms and Temporomandibular Disorders in Healthcare Professionals: The Role of Shift Work, Oral Parafunctions, and Psychological Distress

**DOI:** 10.3390/jcm15135304

**Published:** 2026-07-07

**Authors:** Mehmet Fatih Özsaray, Büşra Özsaray, Elif Pilatin Şahin

**Affiliations:** 1 Department of General Surgery, Kocaeli University, Kocaeli 41001, Turkey; 2 Department of Family Medicine, Kocaeli City Hospital, Kocaeli 41001, Turkey; busrainans@gmail.com; 3 Faculty of Dentistry, Department of Prosthodontics, Department of Clinical Sciences, İstanbul University Cerrahpasa, Istanbul 34015, Turkey; elif.sahin@iuc.edu.tr

**Keywords:** GERD, temporomandibular disorder, shift work, oral parafunction, psychological stress

## Abstract

**Background/Objectives:** Shift work is associated with circadian disruption, sleep disturbances, and psychological distress, all of which may influence both reflux-related symptoms and temporomandibular disorder (TMD)-related complaints. However, the relationships among reflux-related symptom burden, TMD severity, oral parafunctional behaviors, and psychological distress in healthcare professionals remain insufficiently understood. To evaluate the association between reflux-related symptom burden, assessed using the Gastroesophageal Reflux Disease Health-Related Quality of Life (GERD-HRQL) questionnaire, and TMD severity among healthcare professionals, and to investigate the potential roles of shift work, oral parafunctional behaviors, and psychological distress in this relationship. **Methods:** This cross-sectional observational study included healthcare professionals working at a tertiary hospital. Data were collected using validated questionnaires, including the GERD-HRQL, Fonseca Anamnestic Index, Oral Behavior Checklist (OBC), Depression Anxiety Stress Scale-21 (DASS-21), and Epworth Sleepiness Scale (ESS). Participants were categorized as shift workers (≥4 night shifts/month) and non-shift workers. Correlation and multivariable regression analyses were performed. **Results:** A total of 240 participants were included. Higher GERD-HRQL scores were positively correlated with TMD severity (r = 0.31, 95% CI: 0.19 to 0.42, *p* < 0.001), oral parafunctional behavior scores (r = 0.28, 95% CI: 0.16 to 0.39, *p* < 0.001), and DASS-21 stress scores (r = 0.35, 95% CI: 0.23 to 0.46, *p* < 0.001). Shift workers demonstrated significantly higher GERD-HRQL scores and TMD severity scores than non-shift workers, with small-to-moderate effect sizes. In multivariable analysis, higher TMD severity, OBC score, stress score, and shift-work exposure showed adjusted associations with higher GERD-HRQL scores. The model explained 32% of the variance in reflux-related symptom burden (R^2^ = 0.32; adjusted R^2^ = 0.29). **Conclusions**: Higher GERD-HRQL scores, reflecting reflux-related symptom burden rather than objectively confirmed GERD, showed weak to small-to-moderate associations with TMD severity, oral parafunctional behaviors, psychological distress, and shift-work exposure among healthcare professionals. These findings indicate co-occurrence of gastrointestinal, temporomandibular, behavioral, and psychosocial symptom domains within this occupational population. Longitudinal studies using objective diagnostic methods are required to clarify the directionality and clinical significance of these associations.

## 1. Introduction

Gastroesophageal reflux disease (GERD) is a highly prevalent gastrointestinal disorder characterized by troublesome symptoms such as heartburn and regurgitation, which significantly impair quality of life and work productivity [1]. The core pathophysiological mechanisms of GERD include impaired anti-reflux barrier function, transient lower esophageal sphincter relaxations, hiatal hernia, delayed gastric emptying, and impaired esophageal clearance. In addition to these established mechanisms, neural, behavioral, sleep-related, and psychosocial factors may influence symptom perception and perceived symptom severity. In particular, autonomic imbalance, heightened visceral sensitivity, psychological stress, and sleep disturbances have been implicated as modulators of symptom experience rather than primary drivers of GERD pathophysiology [2].

Shift work represents a major occupational stressor that disrupts circadian rhythm and alters physiological homeostasis. Shift work has been consistently associated with impaired sleep quality, hormonal dysregulation, metabolic disturbances, and increased psychological burden [3,4]. Many healthcare professionals are at increased risk of shift-work-related disruption because of frequent night shifts, irregular schedules, and high workloads. Circadian misalignment and sleep fragmentation in this population may not only exacerbate gastrointestinal symptoms but also contribute to broader somatic and functional complaints.

Temporomandibular disorders (TMDs) comprise a group of musculoskeletal conditions affecting the temporomandibular joint and masticatory muscles, typically presenting with pain, joint sounds, and functional limitations [5]. Temporomandibular disorders (TMD) represent a substantial and growing public health burden. Recent evidence suggests that approximately 34% of the population may experience at least one TMD-related symptom, and projections indicate that this proportion may increase to as high as 44% by 2050, highlighting the importance of identifying potentially modifiable behavioral, occupational, and psychosocial factors associated with symptom burden [6]. Current evidence supports a multifactorial biopsychosocial model of TMD, involving biological, psychological, and behavioral influences. Oral parafunctional behaviors, including bruxism and teeth clenching, are considered potential contributing or perpetuating factors rather than isolated primary causes of TMD [7,8]. In addition, contemporary classifications distinguish between sleep bruxism and awake bruxism, which are regarded as related but distinct phenomena with partly different biological and psychosocial determinants. These behaviors are frequently associated with psychological distress, sleep disturbances, and increased pain sensitivity.

Recent evidence has suggested potential overlap in biological, psychological, and behavioral factors associated with both GERD-related symptoms and TMD-related complaints [9,10]. Alterations in autonomic nervous system regulation, stress-related muscle hyperactivity, sleep disturbance, and central sensitization have been proposed as possible contributors to symptom burden in both conditions. In addition, psychological distress has been associated with increased symptom perception in GERD and greater severity of TMD-related complaints [10]. Collectively, these observations suggest that reflux-related symptoms and temporomandibular complaints may frequently coexist in individuals exposed to shared behavioral and psychosocial stressors, although the underlying mechanisms remain incompletely understood.

Despite these observations, the interaction between reflux-related symptoms, TMD severity, oral parafunctional behavior, and psychological distress has not been adequately explored in occupational settings. In particular, healthcare professionals working under shift conditions represent a unique population in whom circadian disruption, stress exposure, and somatic symptom burden may intersect.

Therefore, the aim of the present study was to evaluate the association between reflux-related symptom burden and TMD severity in healthcare professionals and to investigate the potential roles of shift work, oral parafunctional behaviors, and psychological distress in this relationship. We hypothesized that higher reflux-related symptom burden would be associated with greater TMD symptom severity and oral parafunctional behaviors. Because psychological distress and shift-work exposure may act as confounding, mediating, or modifying factors in this relationship, these variables were examined within an exploratory multivariable framework rather than as definitive causal determinants.

## 2. Materials and Methods

### 2.1. Study Design and Setting

This cross-sectional, observational study was conducted at Kocaeli City Hospital, a tertiary referral center. The study was designed in accordance with the Strengthening the Reporting of Observational Studies in Epidemiology (STROBE) guidelines. The study protocol was approved by the Scientific Research Ethics Committee of Kocaeli City Hospital (protocol code: 2025-49; approval date: 3 March 2025). Written informed consent was obtained from all participants.

### 2.2. Study Population

Healthcare professionals aged between 18 and 65 years, who had worked at Kocaeli City Hospital for at least six months, were eligible for inclusion. Participants were recruited through voluntary participation using face-to-face invitations across different clinical departments.

### 2.3. Inclusion and Exclusion Criteria

Additional eligibility criteria were the ability to complete the study questionnaires and provide written informed consent. Individuals were excluded if they were pregnant, had cognitive impairment that could interfere with completion of the questionnaires, had a history of major upper gastrointestinal surgery within the preceding three months, had active malignancy, known neurological disorders, diagnosed psychiatric disorders requiring active treatment, chronic pain syndromes, obstructive sleep apnea, or a history of orthodontic or surgical treatment involving the temporomandibular joint, or declined to participate in the study.

### 2.4. Exposure Definition: Shift Work

Shift work was defined as working ≥ 4 night shifts per month during the preceding month. This threshold was selected to capture regular exposure to circadian disruption rather than occasional or incidental night duty. Previous studies have demonstrated that even a limited number of night shifts per month may adversely affect sleep quality, psychological stress, and circadian alignment in healthcare workers [3,4].

In our setting, healthcare professionals working ≥ 4 night shifts per month are routinely exposed to irregular sleep–wake cycles, nighttime food intake, and repeated circadian disruption. In addition, night shifts are frequently followed by shortened recovery periods or rapid return to daytime duties, which may further exacerbate physiological and psychological stress. Therefore, this definition was considered sufficient to reflect clinically meaningful shift work exposure in a real-world hospital environment.

Participants were categorized into two groups, as shift workers and non-shift workers, based on this definition.

### 2.5. Data Collection and Variables

Data were collected using structured, self-administered, and validated questionnaires, which were completed in a face-to-face setting. Each session lasted approximately 10–15 min. No directly identifiable personal information, such as names or identification numbers, was recorded. All data were anonymized using coded forms to ensure participant confidentiality.

The collected variables included both demographic/occupational characteristics and clinical scale assessments. Demographic and occupational data comprised age, sex, body mass index (BMI), profession and department, duration of employment, monthly number of night shifts, smoking status, daily caffeine consumption, medication use (including proton pump inhibitors and H2 receptor blockers), and any prior diagnosis of GERD.

Reflux-related symptom burden was assessed using the Gastroesophageal Reflux Disease Health-Related Quality of Life (GERD-HRQL) scale, originally developed by Velanovich [11]. The GERD-HRQL was used to quantify reflux-related symptom burden and its impact on health-related quality of life; it was not used to establish a clinical diagnosis of GERD. Temporomandibular disorder severity was assessed using the Fonseca Anamnestic Index. The Fonseca Anamnestic Index was used as a screening instrument for TMD-related symptom severity rather than as a diagnostic reference standard. The Diagnostic Criteria for Temporomandibular Disorders (DC/TMD) remain the current reference standard for clinical diagnosis.

Oral parafunctional behaviors were measured using the Oral Behavior Checklist (OBC), developed by Ohrbach et al. [12]. The OBC assesses self-reported oral behaviors and does not provide a clinical diagnosis of bruxism. Therefore, OBC scores were interpreted as indicators of self-reported parafunctional behavior rather than possible, probable, or definite bruxism. Psychological distress, including depression, anxiety, and stress levels, was evaluated using the Depression Anxiety Stress Scale-21 (DASS-21), originally developed by Lovibond and Lovibond [13]. Daytime sleepiness was assessed using the Epworth Sleepiness Scale (ESS), developed by Johns [14].

All questionnaires were administered in Turkish. Validated Turkish versions were used when available, and the selected instruments have been widely employed in clinical and epidemiological research. Because the study was questionnaire-based, the instruments were interpreted as symptom-screening or self-report measures rather than diagnostic reference standards where applicable.

The primary outcome of the study was reflux-related symptom burden, quantified using the total GERD-HRQL score. Secondary outcomes included TMD severity (Fonseca score), oral parafunctional behavior score (OBC), psychological distress (DASS-21 subscales), and daytime sleepiness (ESS).

### 2.6. Sample Size Calculation

The required sample size was calculated using G*Power, version 3.1.9.7 (Heinrich Heine University Düsseldorf, Düsseldorf, Germany), based on detecting a correlation coefficient of r = 0.20 between GERD-HRQL and TMD severity, with a two-sided α = 0.05 and 80% power. The choice of r = 0.20 was defined a priori and was based on both clinical and methodological considerations. Recent dental research effect-size guidelines classify Pearson’s r of approximately 0.20 as a small effect, whereas values around 0.40 and 0.70 are considered medium and large effects, respectively [15]. Because reflux-related symptoms, TMD-related complaints, oral parafunctional behaviors, psychological distress, and shift-work exposure are multifactorial and symptom-based constructs, small associations were considered clinically plausible and relevant in this exploratory observational setting. Therefore, powering the study to detect r = 0.20 was intended to avoid limiting the analysis only to moderate or large associations.

Based on this assumption, the minimum required sample size was 194 participants. Considering a potential 15–20% rate of incomplete questionnaires or data loss, a total sample size of 240 participants was targeted.

### 2.7. Statistical Analysis

Statistical analyses were performed using IBM SPSS Statistics for Windows, Version 26.0 (IBM Corp., Armonk, NY, USA). The normality of continuous variables was assessed using the Shapiro–Wilk test. Continuous variables are presented as mean ± standard deviation or median with interquartile range (IQR), as appropriate based on distribution. Categorical variables are expressed as frequencies and percentages.

For group comparisons, the independent samples *t*-test was used for normally distributed variables, while the Mann–Whitney U test was applied for non-normally distributed variables. Categorical variables were compared using the chi-square test or Fisher’s exact test when appropriate.

Correlation analyses were performed using Pearson or Spearman correlation coefficients, depending on data distribution, to evaluate the relationships between reflux-related symptom burden and clinical variables, including TMD severity, oral parafunctional behavior, psychological distress, and daytime sleepiness.

To examine variables associated with reflux-related symptom burden after adjustment for selected covariates, a multivariable linear regression model was constructed with GERD-HRQL total score as the dependent variable. Because of the cross-sectional design, regression coefficients were interpreted as adjusted associations rather than independent risk factors, causal predictors, or determinants.

Explanatory variables included Fonseca score, OBC score, DASS-21 stress score, shift-work status, age, sex, BMI, smoking status, and daily caffeine consumption. Multicollinearity among explanatory variables was assessed using the variance inflation factor (VIF), and variables with significant collinearity were excluded from the final model as necessary.

A two-tailed *p*-value of <0.05 was considered statistically significant.

Effect sizes and 95% confidence intervals were reported in addition to *p*-values where appropriate. For normally distributed continuous variables compared using the independent samples *t*-test, Cohen’s d was calculated. For non-normally distributed continuous variables compared using the Mann–Whitney U test, the effect size r was calculated as Z/√N. For categorical comparisons, Cramer’s V was reported where applicable. Correlation coefficients were presented with 95% confidence intervals calculated using Fisher’s z transformation. In the multivariable linear regression model, unstandardized B coefficients, standardized β coefficients, 95% confidence intervals, R^2^, and adjusted R^2^ were reported. Effect-size interpretation was guided by recent dental research recommendations, in which Pearson’s r values of approximately 0.20, 0.40, and 0.70 are considered small, medium, and large effects, respectively [15]. Accordingly, statistical significance was interpreted together with effect size, precision, and clinical relevance.

The assumptions of linear regression, including normality of residuals, homoscedasticity, and linearity, were assessed using residual plots and diagnostic tests.

## 3. Results

A total of 240 healthcare professionals were included in the study, equally distributed between shift workers (*n* = 120) and non-shift workers (*n* = 120). The mean age of the study population was 34.2 ± 7.8 years, and 62.1% of participants were female.

Compared with non-shift workers, shift workers were significantly younger (32.8 ± 7.1 vs. 35.6 ± 8.2 years, *p* = 0.008) and had a shorter duration of employment (*p* = 0.012). Smoking and caffeine consumption were also significantly higher among shift workers (*p* = 0.026 and *p* = 0.001, respectively). Other baseline characteristics, including BMI and medication use, were comparable between the groups (Table 1).

Shift workers demonstrated a significantly higher symptom burden across all evaluated domains. GERD-HRQL scores were significantly higher in shift workers compared to non-shift workers (median 14 vs. 8, *p* < 0.001). Similarly, TMD severity assessed by the Fonseca index was greater in the shift-work group (median 35 vs. 25, *p* = 0.002). Oral parafunctional behavior, psychological distress (all DASS-21 subscales), and daytime sleepiness were also significantly higher among shift workers (all *p* < 0.01).

The proportion of participants with moderate-to-severe TMD was significantly higher among shift workers (46.7% vs. 30.0%, *p* = 0.008), as was the proportion of participants with higher reflux-related symptom burden according to the GERD-HRQL score threshold used in the present study (43.3% vs. 26.7%, *p* = 0.007) (Table 2).

Correlation analysis revealed that reflux-related symptom burden was positively associated with TMD severity (r = 0.31, 95% CI: 0.19 to 0.42, *p* < 0.001), oral parafunctional behavior (r = 0.28, 95% CI: 0.16 to 0.39, *p* < 0.001), and psychological distress, with the strongest correlation observed for stress scores (r = 0.35, 95% CI: 0.23 to 0.46, *p* < 0.001). Daytime sleepiness was also weakly correlated with GERD-HRQL scores (r = 0.22, 95% CI: 0.10 to 0.34, *p* = 0.001). These correlations were weak to small-to-moderate in magnitude according to dental research effect-size recommendations [15].

In multivariable linear regression analysis, higher TMD severity (standardized β = 0.24, *p* < 0.001), higher OBC score (standardized β = 0.15, *p* = 0.021), higher DASS-21 stress score (standardized β = 0.27, *p* < 0.001), and shift work (standardized β = 0.19, *p* = 0.008) were associated with higher GERD-HRQL scores after adjustment for age, sex, BMI, smoking status, and caffeine intake. These findings indicate adjusted associations rather than causal predictors because of the cross-sectional design. The model explained approximately 32% of the variance in reflux-related symptom burden (R^2^ = 0.32; adjusted R^2^ = 0.29) (Table 3). No significant multicollinearity was detected among the explanatory variables (all VIF < 2.5).

## 4. Discussion

In the present study, higher GERD-HRQL scores were associated with greater TMD severity, oral parafunctional behaviors, psychological distress, and daytime sleepiness among healthcare professionals. These associations remained statistically significant after adjustment for selected demographic and occupational variables. In addition, shift-work status was associated with higher GERD-HRQL scores, supporting previous observations that circadian disruption may contribute to increased reflux-related symptom burden in healthcare workers [16,17]. Importantly, in the present study, GERD-HRQL scores should be interpreted as indicators of reflux-related symptom burden and reflux-related quality-of-life impairment, rather than as evidence of objectively confirmed GERD.

The observed co-occurrence of reflux-related symptoms, TMD-related complaints, and oral parafunctional behaviors may reflect overlapping biological, psychological, and behavioral influences. However, because the present study was cross-sectional and relied on questionnaire-based assessments, the findings should be interpreted as evidence of association rather than confirmation of shared underlying mechanisms. This interpretation is consistent with prior evidence suggesting overlap among reflux-related symptoms, shift-work exposure, psychological distress, and oral parafunctional behaviors [16,17,18].

Psychological distress may contribute to both reflux-related symptoms and TMD-related complaints through multiple mechanisms, including altered symptom perception, sleep disturbance, and increased pain sensitivity [16,17,18,19,20]. In our study, stress scores showed the strongest correlation with GERD-HRQL scores. Nevertheless, because only selected psychological variables were included in the analysis, the possibility of residual confounding by other emotional or behavioral factors cannot be excluded.

Oral parafunctional behaviors may represent one of several factors involved in the observed overlap between reflux-related symptom burden and TMD-related complaints. Bruxism, clenching, and repetitive jaw-muscle activity have been linked to psychosocial strain, poor sleep quality, masticatory muscle fatigue, facial pain, and temporomandibular dysfunction [18,21]. In the present study, higher OBC scores were associated with higher GERD-HRQL scores in the multivariable model. However, because the OBC assesses self-reported oral behaviors rather than clinically confirmed bruxism, these findings should be interpreted as associations between self-reported parafunctional behaviors and reflux-related symptom burden, not as evidence of clinically confirmed bruxism or a causal mechanism.

The role of shift work deserves particular attention. Healthcare settings require continuous service delivery, and night work is often unavoidable. Circadian misalignment, irregular meal timing, sleep disturbance, unhealthy behavioral patterns, shortened recovery periods, and psychological stress may contribute to reflux-related symptom burden among shift workers [17,19]. In parallel, sleep disturbance and psychological distress have been associated with increased pain sensitivity, TMD-related complaints, and parafunctional oral behaviors, providing a possible explanation for the broader symptom profile observed among shift workers [18,20,21]. In the present study, shift workers demonstrated higher reflux-related symptom scores, greater TMD symptom severity, higher psychological distress, and greater daytime sleepiness than non-shift workers. Although these findings suggest that shift work is associated with a broader symptom burden, the cross-sectional design cannot determine whether shift work directly contributes to these symptoms or whether other occupational and lifestyle factors account for the observed differences.

Another clinically relevant observation was that the association between GERD-HRQL scores and TMD severity was statistically significant but modest in magnitude. Similarly, the correlations between GERD-HRQL scores and oral parafunctional behaviors, stress scores, and daytime sleepiness were weak to small-to-moderate, and the standardized regression coefficients indicated modest adjusted associations rather than strong predictive or causal effects.

Therefore, the present findings should not be interpreted as evidence that reflux-related symptoms cause TMD-related complaints, or vice versa. Rather, they support the need for further investigation into the complex relationships among reflux-related symptoms, psychosocial factors, occupational stressors, sleep disturbance, and temporomandibular symptoms [12,20].

Our study should also be interpreted in light of its limitations. First, the cross-sectional design does not permit causal inference. Whether reflux predisposes to TMD-related complaints, whether TMD-related distress amplifies reflux perception, or whether both arise from common upstream mechanisms cannot be determined from the present data. Second, all measurements were questionnaire-based. Although validated instruments were used, symptom severity and behavioral exposures were self-reported and therefore susceptible to recall bias and subjective over- or underestimation [11].

Another important limitation is that reflux-related symptoms were assessed using the GERD-HRQL questionnaire rather than objective diagnostic modalities such as upper gastrointestinal endoscopy, esophageal pH monitoring, or impedance testing. Consequently, the present findings should be interpreted as associations involving questionnaire-based reflux-related symptom burden rather than objectively confirmed GERD. Similarly, TMD severity was evaluated using the Fonseca Anamnestic Index, a validated screening instrument rather than a diagnostic reference standard, and oral parafunctional behaviors were assessed through self-reported OBC scores rather than clinically confirmed bruxism.

Third, the study was conducted in a single tertiary hospital, which may limit generalizability to non-healthcare populations or to institutions with different working patterns. Fourth, although the regression model was adjusted for age, sex, BMI, smoking status, caffeine consumption, and shift-work exposure, residual confounding cannot be excluded. Several potentially relevant variables, including dietary habits, sleep duration and quality, alcohol consumption, psychiatric comorbidities, medication use, chronotype, occupational workload, and chronic pain conditions, were not comprehensively assessed and may have influenced the observed associations.

Finally, the multivariable model explained approximately 32% of the variance in GERD-HRQL scores, with an adjusted R^2^ of 0.29, indicating that a substantial proportion of symptom variability remains unexplained and is likely influenced by additional biological, behavioral, occupational, and psychosocial factors not captured in the present study.

Despite these limitations, the study offers several strengths. It addresses a clinically understudied intersection between gastrointestinal, musculoskeletal, behavioral, and psychological symptoms in a real-world occupational cohort. It also applies multiple validated instruments within a single cohort, allowing a more integrated analysis than studies focused on a single symptom domain [11,18].

The predefined shift-work threshold of ≥4 night shifts per month provided a pragmatic measure of regular circadian disruption in this occupational setting and is consistent with previous evidence linking night-shift exposure to impaired sleep quality, circadian misalignment, and psychological stress in healthcare workers [3,4].

The inclusion of shift-work status provides an occupational context for the observed associations, but the findings should not be interpreted as evidence that shift-work modification would directly reduce reflux-related or TMD-related symptoms. Future studies should build on these results using longitudinal designs. Repeated-measures cohorts could clarify whether worsening shift burden or stress levels precede increases in reflux-related symptoms and TMD-related symptoms over time. Studies incorporating actigraphy, sleep diaries, reflux monitoring, or clinical dental examination could also strengthen causal interpretation and reduce reliance on self-report [13,16]. Future interventional studies may also evaluate whether stress management, sleep-focused strategies, or oral habit management are associated with changes in reflux-related and temporomandibular symptom domains.

## 5. Conclusions

In conclusion, in this cross-sectional questionnaire-based study, higher GERD-HRQL scores, representing reflux-related symptom burden rather than objectively confirmed GERD, showed weak to small-to-moderate associations with greater TMD symptom severity, self-reported oral parafunctional behaviors, psychological distress, and shift-work exposure among healthcare professionals.

These findings suggest that gastrointestinal, temporomandibular, behavioral, and psychosocial symptom domains may frequently co-occur within this occupational population. However, because of the cross-sectional design and reliance on questionnaire-based assessments, causal relationships cannot be established. Larger prospective multicenter studies incorporating objective reflux assessment and clinical TMD evaluation are needed to confirm these findings, determine their directionality, and clarify their clinical significance.

## Figures and Tables

**Table 1:** Baseline characteristics of participants according to shift work status.

Variable	Shift Workers	Non-Shift Workers	*p*	Effect Size
Age (years)	32.8 ± 7.1	35.6 ± 8.2	0.008	Cohen’s d = 0.37
Female sex, *n* (%)	79 (65.8)	70 (58.3)	0.231	Cramer’s V = 0.08
BMI (kg/m^2^)	25.1 ± 4.0	26.1 ± 4.6	0.084	Cohen’s d = 0.23
Smoking, *n* (%)	38 (31.7)	23 (19.2)	0.026	Cramer’s V = 0.14
Caffeine (cups/day)	3 (2–4)	2 (1–3)	0.001	Mann–Whitney r = 0.21

Continuous variables are presented as mean ± standard deviation or median (interquartile range) according to distribution; categorical variables are presented as number (%). Effect sizes are reported as Cohen’s d for normally distributed continuous variables, Mann–Whitney r for non-normally distributed continuous variables, and Cramer’s V for categorical variables. BMI: body mass index.

**Table 2:** Comparison of questionnaire-based symptom scores according to shift-work status.

Scale	Shift	Non-Shift	*p*	Effect Size r
GERD-HRQL score	14 (8–21)	8 (4–14)	<0.001	0.33
Fonseca score	35 (25–50)	25 (15–40)	0.002	0.20
OBC score	20 (14–27)	15 (10–22)	0.001	0.21
DASS stress score	10 (6–14)	6 (3–10)	<0.001	0.34
ESS score	9 (6–12)	6 (4–9)	<0.001	0.31

Values are presented as medians with interquartile ranges. Effect size r was calculated for Mann–Whitney U tests as Z/√N. GERD-HRQL: Gastroesophageal Reflux Disease Health-Related Quality of Life; OBC: Oral Behavior Checklist; DASS: Depression Anxiety Stress Scale; ESS: Epworth Sleepiness Scale.

**Table 3:** Multivariable linear regression analysis of factors associated with GERD-HRQL total score.

Variable	B	95% CI for B	Standardized β	95% CI for Standardized β	*p* Value
Fonseca score	0.18	0.09 to 0.27	0.24	0.12 to 0.36	<0.001
OBC score	0.10	0.02 to 0.18	0.15	0.03 to 0.27	0.021
DASS-21 stress score	0.20	0.09 to 0.31	0.27	0.12 to 0.42	<0.001
Shift work (yes vs. no)	2.93	0.88 to 4.97	0.19	0.06 to 0.32	0.008
Age	0.04	−0.04 to 0.12	0.06	−0.06 to 0.18	0.314
Female sex	0.86	−0.91 to 2.64	0.07	−0.07 to 0.21	0.247
BMI	0.13	−0.03 to 0.29	0.09	−0.02 to 0.20	0.118
Smoking	1.48	−0.14 to 3.11	0.11	−0.01 to 0.23	0.089
Daily caffeine consumption	0.39	−0.05 to 0.82	0.10	−0.01 to 0.21	0.104

B: unstandardized regression coefficient; β: standardized regression coefficient; CI: confidence interval; BMI: body mass index; OBC: Oral Behavior Checklist; DASS-21: Depression Anxiety Stress Scale-21. The model was adjusted for age, sex, body mass index, smoking status, and caffeine consumption. R^2^ = 0.32; adjusted R^2^ = 0.29; model *p* < 0.001. Regression coefficients indicate adjusted associations and should not be interpreted as causal predictors because of the cross-sectional design.

## Data Availability

The data presented in this study are not publicly available due to privacy and ethical restrictions, as they contain participant-level clinical and questionnaire data. The data are available from the corresponding author upon reasonable request and subject to applicable ethical approval.

## Abbreviations

The following abbreviations are used in this manuscript:

GERD	Gastroesophageal reflux disease
GERD-HRQL	Gastroesophageal Reflux Disease Health-Related Quality of Life
TMD	Temporomandibular disorder
DASS	Depression Anxiety Stress Scale
BMI	Body mass index
STROBE	Strengthening the Reporting of Observational Studies in Epidemiology
